# Cryostorable callus mimetics support endochondral bone regeneration in a large-animal maxillofacial defect

**DOI:** 10.1093/rb/rbag125

**Published:** 2026-06-10

**Authors:** Flurina Staubli, Kenny Man, Leanne de Silva, Silke Nurmohamed, Nard Janssen, Jan Eelco Bergsma, Alessia Longoni, Debby Gawlitta

**Affiliations:** Department of Oral and Maxillofacial Surgery & Special Dental Care, University Medical Center Utrecht, Utrecht 3584 CX, The Netherlands; Regenerative Medicine Center Utrecht, Utrecht 3584 CT, The Netherlands; Department of Oral and Maxillofacial Surgery & Special Dental Care, University Medical Center Utrecht, Utrecht 3584 CX, The Netherlands; Regenerative Medicine Center Utrecht, Utrecht 3584 CT, The Netherlands; Department of Oral and Maxillofacial Surgery & Special Dental Care, University Medical Center Utrecht, Utrecht 3584 CX, The Netherlands; Regenerative Medicine Center Utrecht, Utrecht 3584 CT, The Netherlands; Department of Oral and Maxillofacial Surgery & Special Dental Care, University Medical Center Utrecht, Utrecht 3584 CX, The Netherlands; Department of Oral and Maxillofacial Surgery & Special Dental Care, University Medical Center Utrecht, Utrecht 3584 CX, The Netherlands; Department of Oral and Maxillofacial Surgery & Special Dental Care, University Medical Center Utrecht, Utrecht 3584 CX, The Netherlands; Regenerative Medicine Center Utrecht, Utrecht 3584 CT, The Netherlands; Department of Orthopedics, University Medical Center Utrecht, Utrecht 3584 CX, The Netherlands; Department of Oral and Maxillofacial Surgery & Special Dental Care, University Medical Center Utrecht, Utrecht 3584 CX, The Netherlands; Regenerative Medicine Center Utrecht, Utrecht 3584 CT, The Netherlands

**Keywords:** endochondral bone regeneration, maxillofacial bone reconstruction, devitalized cartilage matrix, mesenchymal stromal cells, large animal model

## Abstract

Endochondral bone regeneration (EBR) is a developmentally inspired strategy based on a pre-formed cartilage template that remodels in vivo into vascularized bone. Although EBR has shown promise in orthopedic settings, its application to maxillofacial reconstruction remains largely unexplored in large animal models, particularly because maxillofacial bone is classically formed by intramembranous ossification. Here, we present soft callus mimetics (CMs) as bioactive, devitalized cartilage modules that preserve extracellular matrix-encoded cues capable of instructing endochondral ossification without viable donor cells. Their regenerative performance was evaluated in a split-mouth bilateral goat alveolar cleft model (n = 10), in which CMs were implanted in one cleft and autologous iliac crest bone graft in the contralateral cleft. Across microCT, histomorphometry, and fluorochrome labeling, CM-treated defects supported bone regeneration and remodeling without evidence of inferior early structural outcomes compared with autograft. In a separate rat subcutaneous implantation study, CMs retained osteoinductive capacity after 9 months of cryostorage compared with previously reported fresh CMs implanted under matched, non-concurrent conditions. Together, these findings support the biological feasibility of CM-driven endochondral bone regeneration in a large animal maxillofacial environment and the further development of cryostorable CMs for bone repair.

## Graphical abstract

**Graphical Abstract:**
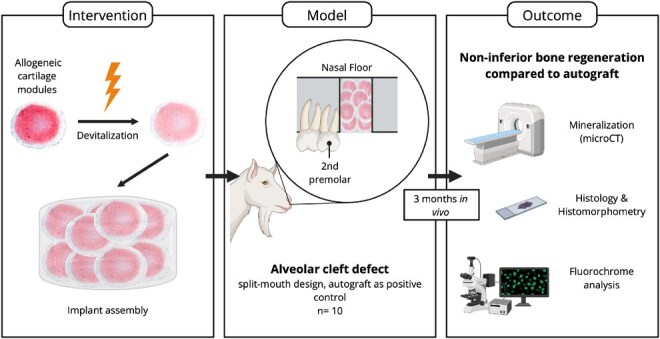

## Introduction

Endochondral bone regeneration (EBR) has emerged as a powerful strategy in bone tissue engineering. This approach leverages the intrinsic sequence of endochondral ossification that underlies long bone development and fracture healing [1]. By establishing a transient cartilage template that remodels into vascularized bone *in vivo*, EBR can offer advantages over direct intramembranous approaches, particularly by supporting vascular invasion and coupling matrix remodeling to new bone formation in large defects [2–4]. Robust bone regeneration has been demonstrated in preclinical orthopedic models using chondrogenically primed or devitalized cartilage-based constructs [5–7], and encouraging results have also been reported in rodent cranial [8, 9] and maxillofacial [10] defect models.

However, the potential of EBR for maxillofacial reconstruction remains insufficiently defined, especially in translationally relevant large animal settings [11]. Maxillofacial bones form predominantly through intramembranous ossification and derive largely from neural crest lineages, in contrast to the mesoderm-derived long bones that typically regenerate via endochondral pathways. These developmental distinctions are reflected in adult tissue properties, including differences in vascular architecture, progenitor cell identity, and molecular responses to osteogenic cues [12, 13]. In craniofacial sites such as the calvarium, these features can result in slower remodeling and altered responsiveness to osteogenic stimuli compared with long bones [14–18]. This underscores the need to evaluate regeneration strategies in the anatomical context for which they are intended. To date, most craniofacial EBR studies have used small calvarial defects in rodents [8, 9]. Although these defects are developmentally related to maxillofacial bones, they differ anatomically and mechanically. Only a limited number of studies have investigated EBR-based constructs as graft extenders [19] or implants [10] in mandibular defects. Moreover, these models do not recapitulate the three-dimensional geometry and functional demands typical of clinical maxillofacial reconstruction. As a result, it remains unclear whether cartilage-template-driven regeneration can reliably produce robust, integrated bone in a large animal maxillofacial environment.

Our group previously developed soft callus mimetics (CMs): devitalized cartilage constructs derived from allogeneic mesenchymal stromal cells (MSCs) that are chondrogenically differentiated to emulate the soft-callus stage of fracture repair. Our mild devitalization process preserves extracellular matrix structure and matrix-associated pro-regenerative cues while reducing cellular immunogenicity [5, 6, 20]. Beyond serving as a single implant concept, CMs represent bioactive, cartilage-derived ECM units that can function as modular building blocks for endochondral regeneration. Their malleable and cohesive format allows them to be shaped, packed, or combined to match complex defect geometries. At the same time, they retain instructive cues that support vascular invasion, matrix remodeling, and subsequent bone formation. Consistent with this concept, CMs have supported robust endochondral bone regeneration in long-bone defects in both small- and large animal models [5, 6]. Importantly, the devitalization protocol includes a lyophilization step, creating implants intended to be compatible with frozen storage. This may offer practical advantages over living or freshly prepared biologic grafts, which require maintenance of cell viability and typically have limited storage windows. However, this proposed long-term stability has not yet been experimentally validated, and the impact of prolonged storage on *in vivo* regenerative performance remains unknown.

In this study, we evaluated whether a CM-based endochondral approach can drive bone regeneration in a maxillofacial environment. CMs generated from two independent goat MSC donors were first characterized for chondrogenic matrix formation and pro-regenerative markers before being combined into a single implant formulation for *in vivo* testing. A bilateral goat alveolar cleft model was used as a surgically relevant large animal defect that enabled direct within-animal comparison with autologous iliac crest bone grafting. Each animal received a CM implant in one cleft and an autologous graft in the contralateral cleft, allowing assessment of bone formation, defect bridging and tissue integration. In parallel, a complementary rat subcutaneous implantation study was performed to test whether CMs retained osteoinductive capacity after prolonged cryostorage. This addressed storage stability as a key translational requirement for a future off-the-shelf format. Because this was designed as a first large animal proof-of-concept study, the primary aim was to evaluate the feasibility and biological performance of CM-driven endochondral regeneration in a maxillofacial defect, rather than to establish definitive clinical superiority over autograft. We hypothesized that CMs would support bone regeneration comparable to autografts in the maxillofacial defect. Such a finding would show that endochondral regeneration can be extended beyond orthopedic applications. It would also support the development of CMs as a scalable, storable implant format for future clinical translation.

## Materials and methods

### Study design and overview

This work comprised two complementary phases. First, a goat maxillofacial proof-of-concept study was performed to evaluate whether CM-driven endochondral bone regeneration can support bone formation in a maxillofacial defect with an intramembranous developmental origin. Second, a rat subcutaneous study was performed to assess whether CMs retain osteoinductive capacity after prolonged cryostorage. All animal procedures complied with the ARRIVE guidelines [21] and were approved by the Central Authority for Scientific Procedures on Animals (Dutch national CCD) and the Local Animal Welfare Body (rat protocol no.: 2465-1-02; goat protocol no.: 8831-1-01).

**Figure 1:**
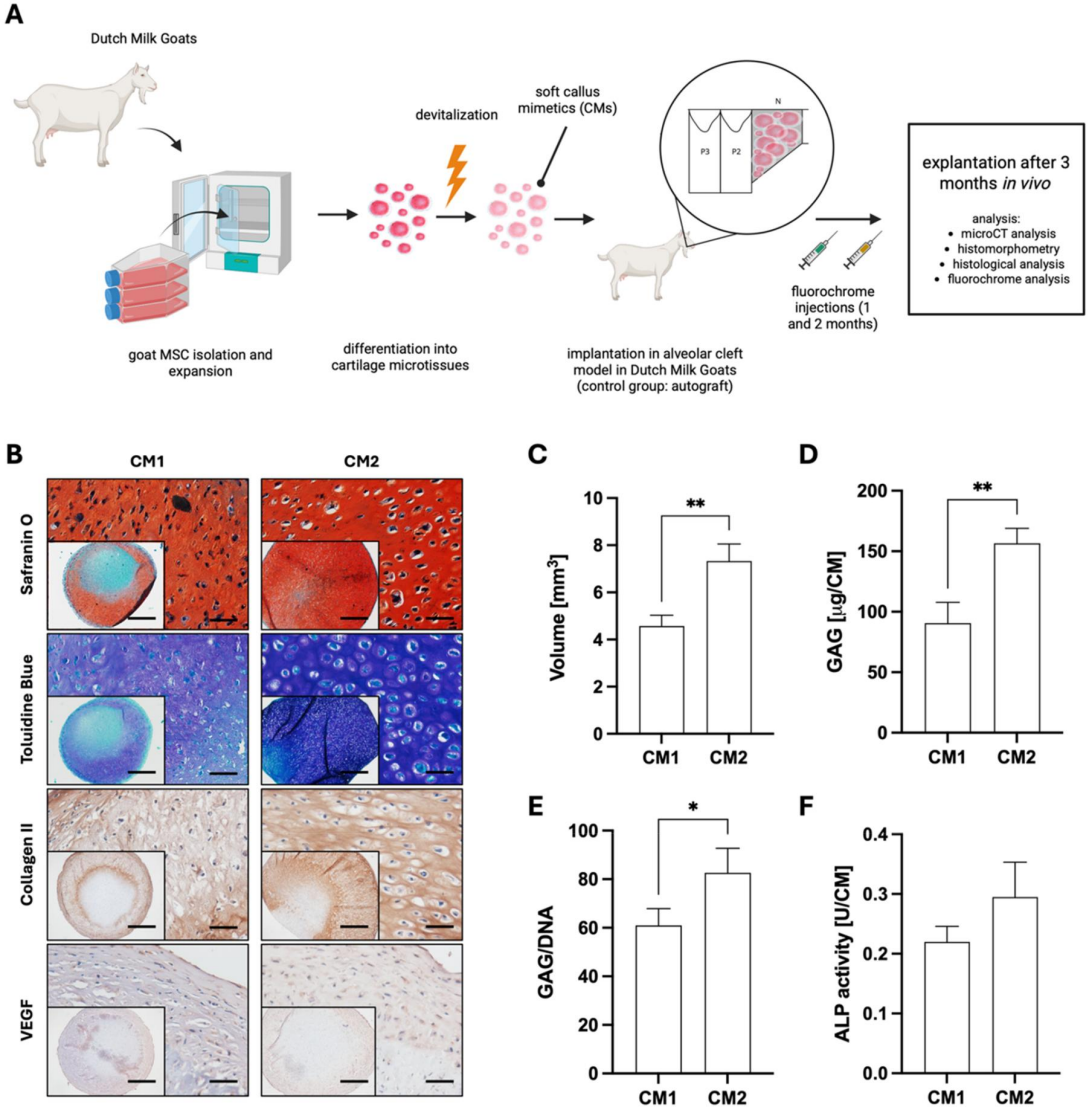
Characterization of goat soft callus mimetics (CMs) derived from two independent MSC donors (CM1 and CM2). (**A**) schematic overview of the bilateral goat alveolar cleft model, in which devitalized goat MSC‑derived CMs were implanted in one cleft and an iliac crest autograft in the contralateral cleft. Calcein green and oxytetracycline fluorochrome labels were administered at 1 and 2 months, respectively, and bone regeneration was analyzed after 3 months. (**B**) Representative sections stained with safranin O/fast green, toluidine blue/fast green, and immunostainings for collagen II and VEGF. Insets show low-magnification overviews. Scale bars: 50 μm (main) and 500 μm (insets). Corresponding mouse isotype controls for collagen II and VEGF immunostaining are shown in Supplementary Figure S2. (**C**) CM volume. (**D**) Total GAG content per CM. (**E**) GAG/DNA ratio. (**F**) Alkaline phosphatase (ALP) activity per CM. Data are presented as mean ± SD; *P < 0.05, **P < 0.01. *N* = 4.

In the goat study, CMs derived from allogeneic goat MSCs (gMSCs) were implanted in a bilateral alveolar cleft model to test the feasibility of EBR in a large animal maxillofacial bone defect. Each animal received CMs in one cleft and an autologous iliac crest graft (AG) in the contralateral cleft, enabling within-animal comparison of bone formation and integration. Calcein green and oxytetracycline were administered at 1 and 2 months post-implantation, respectively, to track dynamic bone formation. After 3 months, bone regeneration was assessed by microCT, histology, histomorphometry and fluorochrome analysis (Figure 1A).

Building on these findings, a rat subcutaneous implantation study was conducted to evaluate the long-term storage stability of CMs as a translational proof-of-concept. Stored constructs generated from rat MSCs (rMSCs; Dark Agouti rats) were implanted subcutaneously after 9 months of dry storage at −80°C. This storage period was selected as a prolonged proof-of-concept interval to assess whether frozen storage affects retained osteoinductive capacity. Bone formation after 12 weeks was analyzed by microCT, histology, and histomorphometry, and compared with previously reported fresh CMs generated and implanted under matched experimental conditions [20] (Figure 5A).

### Goat study

#### Generation of goat CMs

gMSCs were isolated from iliac wing biopsies of adult female Dutch milk goats (Capra hircus, *N* = 2) as previously described [5]. Cells from the two independent goat donors were processed separately throughout expansion, chondrogenic differentiation, and devitalization; the resulting CM batches are referred to as CM1 and CM2 throughout the manuscript. Briefly, bone marrow was flushed with MSC expansion medium composed of α–minimum essential medium (α-MEM; 12571063, Thermo Scientific) supplemented with 10% heat-inactivated fetal bovine serum (S14068S1810, Biowest), 0.2 mM L-ascorbic acid 2-phosphate (A8960, Sigma‐Aldrich), and 100 U/mL penicillin with 100 μg/mL streptomycin (15140, Invitrogen). Bone fragments were removed using a 70 µm cell strainer, and adherent cells were expanded under standard conditions. Cultures were passaged at 80% confluency until passage 4 and frozen until further use.

The adipogenic and osteogenic differentiation capacity of both goat MSC donor-derived cell populations was confirmed before CM generation, with results shown in Supplementary Figure S1. For differentiation assays, passage 4 gMSCs were seeded in monolayer culture at 2500 cells/cm^2^ for adipogenic differentiation and 1500 cells/cm^2^ for osteogenic differentiation, and initially maintained in MSC expansion medium. After 2 days, the medium was replaced with either adipogenic or osteogenic differentiation medium. Corresponding control cultures were maintained in MSC expansion medium for the same duration. Osteogenic differentiation medium consisted of standard expansion medium supplemented with 10 mM β-glycerophosphate (G9891; Sigma-Aldrich) and 10 nM dexamethasone (D8893; Sigma-Aldrich). Adipogenic differentiation medium consisted of standard expansion medium without L-ascorbic acid 2-phosphate, supplemented with 1 μM dexamethasone (D8893; Sigma-Aldrich), 0.5 mM 3-isobutyl-1-methylxanthine (I5879; Sigma-Aldrich), 0.2 mM indomethacin (17378; Sigma-Aldrich) and 1.72 μM insulin (I0516; Sigma-Aldrich). Both differentiation media were refreshed twice per week. After 10 days of differentiation, osteogenic differentiation was assessed by alkaline phosphatase staining (ab64254; Abcam; 20 min incubation), and adipogenic differentiation was assessed by Oil Red O staining (O0625; Sigma-Aldrich; 20 min incubation).

Cartilaginous spheroids were generated from passage 4 gMSCs according to established protocols [5]. Briefly, gMSCs (20 × 10^6^ cells/mL) were encapsulated in type I collagen hydrogel (4 mg/mL; 354249, Corning; prepared according to the supplier’s instructions). Approximately 1 × 10^6^ cells in 50 µL hydrogel were dispensed into 96-well round-bottom plates, allowed to polymerize at 37°C for 30 min, and subsequently transferred to 48-well plates containing chondrogenic differentiation medium. The medium consisted of high-glucose Dulbecco’s modified Eagle medium (DMEM; 10566016, Thermo Fisher Scientific), 1% ITS (insulin‐transferrin‐selenium)+ premix (354352, Corning), 100 nM dexamethasone (D8893, Sigma‐Aldrich), 0.2 mM L‐ascorbic acid 2‐phosphate, 100 U/mL penicillin with 100 μg/mL streptomycin (15140, Invitrogen), 10 ng/mL transforming growth factor‐β1 (TGFβ1; Peprotech, New Jersey, USA) and 100 ng/mL bone morphogenetic protein‐2 (BMP‐2; InductOS, Wyeth/Pfizer). Spheroid formation occurred over 28 days, with medium changes performed daily for the first four days and every three days thereafter. After 28 days of chondrogenic differentiation, spheroids were devitalized using a mild protocol involving lyophilization as previously described [6], yielding the final CM building blocks for implant fabrication.

#### Characterization of goat CMs

Prior to implantation, goat CMs were characterized for chondrogenic matrix deposition, protein expression, and biochemical activity.

For histological characterization, CMs were washed with PBS and fixed overnight with 4% formaldehyde. Following fixation, samples were dehydrated through a graded ethanol series (70–100%), cleared in xylene, and embedded in paraffin. Sections of 5 µm thickness were prepared using a microtome (Microm HM340E; Thermo Fisher Scientific). Chondrogenic matrix deposition was qualitatively assessed using Safranin O/Fast Green and Toluidine Blue/Fast Green staining to visualize glycosaminoglycans (GAGs) as previously described [5, 6, 20]. For Safranin O/Fast Green staining, sections were deparaffinized, washed with deionized water, and stained with Weigert’s hematoxylin. After incubation in running tap water, sections were rinsed with deionized water and stained with 0.4% w/v Fast Green (F7252, Sigma-Aldrich) to visualize collagenous matrix. After washing with 1% v/v acetic acid, sections were incubated with 0.125% w/v Safranin O (TMS-009, Sigma-Aldrich) to visualize proteoglycans. For Toluidine Blue/Fast Green staining, deparaffinized and rehydrated sections were stained with 0.4% Toluidine Blue solution (T3260, Sigma-Aldrich) in 0.1 M sodium acetate buffer (pH 4; Sigma-Aldrich) to visualize proteoglycans and counterstained with 0.2% w/v Fast Green (F7252, Sigma-Aldrich). Stained sections were dehydrated through 96% and 100% ethanol, cleared in xylene, mounted in Eukitt Quick-hardening mounting medium (03989, Sigma-Aldrich).

Immunohistochemical staining was performed to visualize collagen type II protein and vascular endothelial growth factor (VEGF) protein expression. Endogenous peroxidase activity was blocked by incubation with 0.5% v/v hydrogen peroxide. For collagen type II staining, antigen retrieval was performed by sequential incubation with 1 mg/mL pronase and 1 mg/mL hyaluronidase for 30 min each at 37°C. For VEGF staining, antigen retrieval was performed by heating sections in 10 mM citrate buffer (pH 6) at 95°C for 20 min. Non-specific protein binding was blocked with 5% w/v bovine serum albumin in PBS for 30 min at room temperature. Collagen type II primary antibody (II‐II6B3) concentration was 0.6 µg/mL and VEGFA primary antibody (VG‐1; ab1316) concentration was 5 µg/mL. BrightVision HRP‐anti‐mouse IgG (VWRKDPVM110HRP) was used as a secondary antibody for 30 min at RT. The staining (brown) was developed by 3,3′‐diaminobenzidine (DAB) oxidation. To visualize the nuclei, sections were counterstained with hematoxylin. Lastly, the slides were dehydrated and mounted with Eukitt Quick‐hardening mounting medium (Sigma-Aldrich). A mouse isotype control (X0931, Dako) was used as a negative control at a concentration matched to the corresponding primary antibody and is shown in Supplementary Figure S2.

All sections were visualized using an Olympus BX51 microscope (Olympus DP73 camera, Olympus).

Biochemical assays were conducted to quantify chondrogenic differentiation. CMs were digested overnight at 60°C in papain digestion buffer (250 µg/mL papain, 0.2 M NaH_2_PO_4_, 0.1 M EDTA, and 0.01 M DL-cysteine hydrochloride; Sigma-Aldrich). Total GAG content was determined by a 1,9-dimethyl-methylene blue (DMMB, pH 3.0; Sigma-Aldrich) assay, with chondroitin sulfate C (Sigma-Aldrich) used as a standard. Absorbance was recorded at 525 and 595 nm. DNA content was determined using the Quant-iT PicoGreen assay according to the manufacturer’s instructions, with fluorescence measured at 485 nm excitation and 535 nm emission, and quantified against a DNA standard curve. GAG content was normalized to DNA content (DMMB/DNA).

Alkaline phosphatase (ALP) activity was measured using the p-nitrophenyl phosphate (pNPP) substrate system (N2765; Sigma-Aldrich). Known concentrations of ALP (U/min/mL) were included to generate a standard curve. CMs and standards were incubated individually with pNPP substrate at 37°C for 5 min. Absorbance was measured at 405 nm with a reference wavelength of 655 nm.

#### Assembly of implants

Fibrin was isolated from allogeneic goat blood (collected in heparin-coated Vacutainer tubes) using the Vivostat Fibrin Preparation Kit (Vivostat, VS306) and processed with the Vivostat Processor Unit according to the manufacturer’s instructions. Fibrin was stored at −20°C until use. A total of 70 CMs (35 CM1 and 35 CM2 constructs) were embedded within the allogeneic fibrin matrix, resulting in a 1:1 donor ratio. This pooled formulation was selected before *in vivo* implantation to reduce dependence on a single donor-derived CM batch and to generate one standardized allogeneic implant formulation for the goat study. The fibrin matrix was manually crosslinked using a pH 4 solution, reaching a total implant volume of approximately 1.5 cm^3^. Because the CMs had been devitalized before fibrin embedding, donor-cell viability was not affected by this crosslinking step. Implants were prepared in the morning before surgery and kept in PBS until implantation to minimize prolonged exposure to the acidic crosslinking solution.

#### Alveolar cleft defect model

Bilateral alveolar cleft defects were created in adult Dutch milk goats (Capra hircus; *n* = 10; females; age > 3 years; weight range 60–90 kg). In addition to alveolar cleft creation, the animals also received alveolar augmentation defects, iliac crest defects and intramuscular implantations as part of parallel studies. All animals were acclimated for at least one week prior to surgery. Animals were fed with pre-moistened chow (standard chow, beets and grass pulp) from 1 week prior to surgery until up to 1 week post-surgery.

All procedures were performed under general anesthesia. Anesthesia was induced with detomidine (intramuscular, 0.04 mg/kg) and propofol (intravenous, 2 mg/kg), and maintained with continuous intravenous infusion of propofol (10 mg/kg/h), cisatracurium (0.09 mg/kg/h), and sufentanil (0.003 mg/kg/h). Preoperative analgesia consisted of a transdermal buprenorphine patch (35 µg/h), and intraoperative analgesia was supplemented with meloxicam (0.5 mg/kg, intravenous). Postoperative analgesia was provided with the buprenorphine patch (35 µg/h) for three days. Antibiotic prophylaxis included amoxicillin–clavulanate potassium (10 mg/kg, intravenous) administered immediately prior to surgery, followed by intramuscular penicillin (10 000 IE/kg) and streptomycin (10 mg/kg) for five consecutive days, including the day of surgery.

For surgery, animals were placed in sternal recumbency. Following extraction of the left and right maxillary first premolars, buccal and palatal mucoperiosteal flaps were elevated. Bilateral three-wall bony defects (∼1.5 cm^3^) were created by removal of buccal, palatal, and nasal bone, while preserving the nasal mucosal layer. Before implant placement, a 1 × 1 × 1 cm piece of Spongostan (Ethicon, USA) was pressed flat and positioned along the nasal floor margin of each defect to prevent leakage from the nasal cavity into the defect. One defect was reconstructed with CMs embedded in allogeneic fibrin, whereas the contralateral defect was grafted with autologous cancellous iliac crest bone chips. Treatment allocation to the left or right defect was randomized before surgery. The mucoperiosteal flaps were closed in a tension-free manner with resorbable sutures (Vicryl 3-0, Ethicon).

Calcein green (10 mg/kg in 2% w/v NaHCO_3_) and oxytetracycline (16 mg/kg) were administered intravenously at 1 and 2 months postoperatively, respectively. Animals were euthanized 3 months post-surgery by intravenous overdose of pentobarbital (to effect). Following euthanasia, the upper jaws were harvested, rinsed in PBS, and fixed in 4% w/v formaldehyde for further analysis.

#### MicroCT scanning and analysis

After explantation, the samples were scanned using a Quantum FX microCT system (Perkin Elmer, Waltham, MA, USA) with the following scan settings: field of view (FOV): 30 mm, voltage: 90 kV, current: 160 µA and scan time: 3 min. Reconstructed three-dimensional images were generated using ImageJ software. To evaluate mineralized tissue formation within the defect region, a grayscale intensity threshold was applied to distinguish mineralized from non-mineralized tissue. The same threshold was applied to all samples to allow direct comparison between treatment groups. Each dataset was reoriented to a standardized anatomical plane, with coronal slices aligned symmetrically through the center of the second premolars bilaterally. The starting point for analysis was defined as the first coronal slice immediately anterior to the second premolar, corresponding to the posterior boundary of the defect. From this reference slice, 60 consecutive slices were analyzed in the anterior direction toward the snout. Mineralized volume within this defined region of interest was quantified to assess bone regeneration using the BoneJ plugin in ImageJ.

#### MMA section preparation

Following fixation, samples were dehydrated through a graded ethanol series (70, 90, 95 and 100% v/v; 24 h per step) and embedded in methylmethacrylate (MMA). Excess bone surrounding the defect was trimmed to approximately the defect margins using an automated sandpapering system. Sagittal plane sections (50 µm thickness) were obtained using a diamond-coated hard tissue microtome (SP 1600, Leica Microsystems). Sections were stained with methylene blue and basic fuchsin for histological and histomorphometric evaluation. Briefly, sections were incubated in 1% acid alcohol, prepared from 99.5% ethanol and 37% hydrochloric acid (100317, Merck), for 1 min and rinsed twice with deionized water. Sections were then stained for 30 s with 1% w/v methylene blue (M9140, Sigma-Aldrich) in borax buffer (1.06045, Sigma-Aldrich), pH 8.5, rinsed twice with deionized water, and subsequently stained for 30 s with 0.3% w/v basic fuchsin (80056, Klinipath) in deionized water. After final rinsing, sections were dried and mounted for bright-field imaging. This staining enabled evaluation of mineralized and non-mineralized tissues within the defect region, with nuclei appearing blue, soft tissues/muscle pink to red and bone/osteoid bright pink. Unstained sections were kept for fluorescence-based fluorochrome analysis.

#### Quantitative and qualitative assessment of bone formation

Overview images of sagittal methylene blue/basic fuchsin-stained MMA sections through the central defect plane were acquired using a Thunder imaging system (Leica Microsystems, Germany). The defect region was manually outlined and designated as the region of interest (ROI). Within this ROI, bone areas were manually segmented using the quick selection tool in Adobe Photoshop 23.2.2. Pixel counts for each segmented area were obtained using the ‘Record Measurement’ function in the Measurement Log. The bone content was expressed as a percentage of the total ROI cross-sectional area. All analyses were performed in a blinded manner and the mean value obtained from two central slides was used for statistical analysis.

Dynamic bone formation was assessed using fluorochrome labeling. One central unstained MMA section per defect was imaged with a confocal microscope (Leica DMi8) using a 10× objective. Calcein green was detected at excitation 495 nm/emission 515 nm, and oxytetracycline was detected at excitation 380 nm/emission 510 nm. For qualitative scoring of fluorochrome distribution, three standardized regions (Figure 4A) were assessed in each defect: the defect center (1), the side edge (2) and the bottom edge (3). For each region, calcein green and oxytetracycline labeling were scored separately as binary outcomes. A region was scored as ‘fully labeled’ when the fluorochrome signal was distributed throughout the bone area within that region. If part of the bone area lacked fluorochrome signal, the region was scored as ‘not fully labeled’. The resulting binary scores were summarized as heat maps showing the proportion of animals with fully labeled bone in each region and treatment group. Scoring was performed in a blinded manner.

### Rat study

#### Generation of rat CMs

rMSCs were isolated, expanded, chondrogenically differentiated, and devitalized as part of, and as described in, a previous study [20]. MSC identity and trilineage differentiation capacity of rMSCs from the same original isolation were characterized in a separate previous study [22]. In the previous study [20], freshly prepared CMs from this production run were implanted subcutaneously in collagen gel to establish baseline regenerative performance. For the present study, additional CMs from the same production run were stored dry for 9 months at −80°C and then implanted under matched conditions, including collagen carrier, implantation site, recipient rat strain, implantation duration, and analytical endpoints. Fresh CM data used for comparison were obtained from the previous study [20] and were not generated concurrently with the stored CM group.

#### Subcutaneous implantation model

This study replicated the subcutaneous implantation conditions of our previous experiment using fresh CMs, but here only stored implants were placed *in vivo*. The stored CM group was matched to the previously reported fresh CM group for key experimental parameters [20]. These included CM production run, collagen carrier, implantation site, recipient rat strain, implantation duration, and analytical endpoints. Fresh CM data were obtained from the earlier study and were not generated concurrently with the stored CM group. Eight Brown Norway rats (10–12 weeks old, 200–250 g) were housed in pairs under standard laboratory conditions (12 h light/12 h dark cycle, 21°C) with *ad libitum* access to water and standard chow. Animals were acclimated for one week prior to surgery.

Each rat received two subcutaneous pockets, permitting implantation of two CM constructs per animal. In each pocket, two stored CMs were encapsulated in rat tail type I collagen hydrogel (4 mg/mL; 354249, Corning; prepared according to the supplier’s instructions) before implantation. Across all animals, 16 implants were placed (*n* = 16).

Anesthesia was induced with 4% isoflurane and maintained at 1.5% isoflurane. Analgesia was administered subcutaneously using buprenorphine (0.05 mg/kg) and carprofen (5 mg/kg); a single prophylactic dose of Duplocilline LA (22 000 IE/kg) was provided. Carprofen (5 mg/kg) was additionally administered once daily for three days postoperatively. Surgical sites were shaved and disinfected with Betadine. Skin incisions were made with a scalpel, subcutaneous pockets were created by blunt dissection with curved scissors, CM constructs were inserted, and the incisions were closed with 4-0 Vicryl resorbable sutures. Animals were euthanized 12 weeks post-implantation; implants were retrieved for analysis, rinsed in PBS, and fixed in 4% (w/v) formaldehyde. Implants that could not be retrieved were excluded from subsequent analysis. Of the 16 stored implants placed, 9 were retrieved and included in the final analysis.

#### MicroCT scanning and analysis

Mineralization was assessed using a Quantum FX microCT system (PerkinElmer). Each specimen was scanned for 3 min with an isotropic voxel size of 42 µm under the following parameters: 90 kV, 160 µA, and field of view (FOV) = 21 mm. A global threshold for mineralized tissue was applied, and mineralized volume was quantified using the BoneJ plugin (v2.9.0) in ImageJ. The same thresholding approach as used for the previously reported fresh CM group [20] was applied to enable comparison between fresh and stored constructs.

#### Histological processing and histomorphometry

Following microCT, specimens were decalcified in 10% EDTA (pH 7.4) for two weeks, dehydrated through a graded ethanol series, cleared in xylene, and embedded in paraffin. Paraffin blocks were sectioned at 5 μm. Hematoxylin and eosin (H&E) staining and Safranin O/Fast Green staining were performed on rehydrated sections as previously described [6, 20]. For H&E staining, sections were deparaffinized, rehydrated through a graded ethanol series, stained with hematoxylin (1.09249.0500, Merck), differentiated and blued, counterstained with 1% w/v eosin (1.15935.0025, Merck), dehydrated, cleared in xylene, and mounted in Eukitt Quick-hardening mounting medium (03989, Sigma-Aldrich). Safranin O/Fast Green staining was performed as described above to visualize proteoglycan-rich cartilaginous matrix and surrounding collagenous tissue. Sections were imaged using an Olympus BX51 microscope equipped with an Olympus DP73 camera. Overview images were generated using the automated photomerge function in Adobe Photoshop 2022. Histomorphometric analysis was performed on H&E-stained sections from one central slide per explant to quantify bone, bone marrow, and cartilage, expressed as percentages of the total CM area. Quantification was performed in a blinded manner.

### Statistics

All data are presented as mean ± standard deviation (SD). For the comparison between CM1 and CM2, unpaired t-tests were conducted. For the goat alveolar cleft study, within-animal comparisons between CM and AG sites were analyzed using a paired one-sided t-test (alternative hypothesis: CM < AG). This one-sided test was selected because the predefined statistical question was whether CM-treated defects showed inferior bone formation compared with the autograft control. To assess the relative contribution of treatment and inter-animal variability, a two-way ANOVA was performed with animal as the row factor and treatment as the column factor; variance components were expressed as a percentage of total variation. For the rat subcutaneous study, group comparisons between fresh and stored CMs were performed using an unpaired one-sided t-test to assess whether stored CMs showed lower bone-forming capacity than freshly implanted CMs. Statistical significance was defined as *P* < 0.05. All analyses were conducted using GraphPad Prism (version 10.6.1; GraphPad Software, San Diego, CA, USA).

## Results

### Endochondral bone regeneration in a maxillofacial model

In a bilateral alveolar cleft model in goats, CMs derived from allogeneic gMSCs were compared with contralateral iliac crest autografts to test whether EBR can support maxillofacial bone regeneration. Each animal received a CM-based graft in one cleft and an autograft in the other. All animals were euthanized after 3 months, and bone healing was assessed by microCT, histology, histomorphometry, and fluorochrome labeling following calcein green (1 month) and oxytetracycline (2 months) administration. An overview of the experimental workflow is depicted in Figure 1A.

#### Characterization of soft callus mimetics

Goat CMs were generated from multipotent mesenchymal stromal cells (MSCs) of two independent allogeneic goat donors (CM1 and CM2). Safranin O/Fast Green and Toluidine Blue/Fast Green staining confirmed successful chondrogenic differentiation and robust glycosaminoglycan (GAG) deposition in chondrogenic spheroids cultured from both donor groups (red and dark blue/purple, respectively). Notably, GAG accumulation in CM1 was predominantly peripheral, whereas CM2 showed a more uniform distribution. Collagen type II immunostaining further reflected these donor-specific differences, mirroring GAG localization patterns (Figure 1B). Quantitatively, CM2 constructs were larger than CM1 constructs (7.33 ± 0.73 mm³ vs. 4.58 ± 0.46 mm³; Figure 1C), and contained more GAGs (156.50 ± 12.48 µg vs. 90.05 ± 17.31 µg; Figure 1D), with a correspondingly higher GAG/DNA ratio (82.63 ± 10.07 vs. 60.92 ± 6.96; Figure 1E). Despite these differences in matrix deposition, both CM batches demonstrated successful chondrogenic maturation and substantial extracellular matrix deposition. Both batches also showed pro-angiogenic and osteogenic components, including VEGF (Figure 1B) and ALP activity (0.30 ± 0.06 vs. 0.22 ± 0.03 U/CM; Figure 1F). Thus, CMs from both donors were considered suitable for implantation and were combined in a 1:1 ratio as a pooled allogeneic implant formulation for use *in vivo*.

#### Surgical handling and healing

Fibrin-embedded CM implants were easily adapted to defect contours and maintained structural coherence during manipulation. The constructs remained stable within the cleft site throughout placement and closure (Supplementary Figure S3). Postoperatively, all surgical sites healed uneventfully, with no wound dehiscence, infection, or other adverse events observed.

#### Mineralized volume

**Figure 2:**
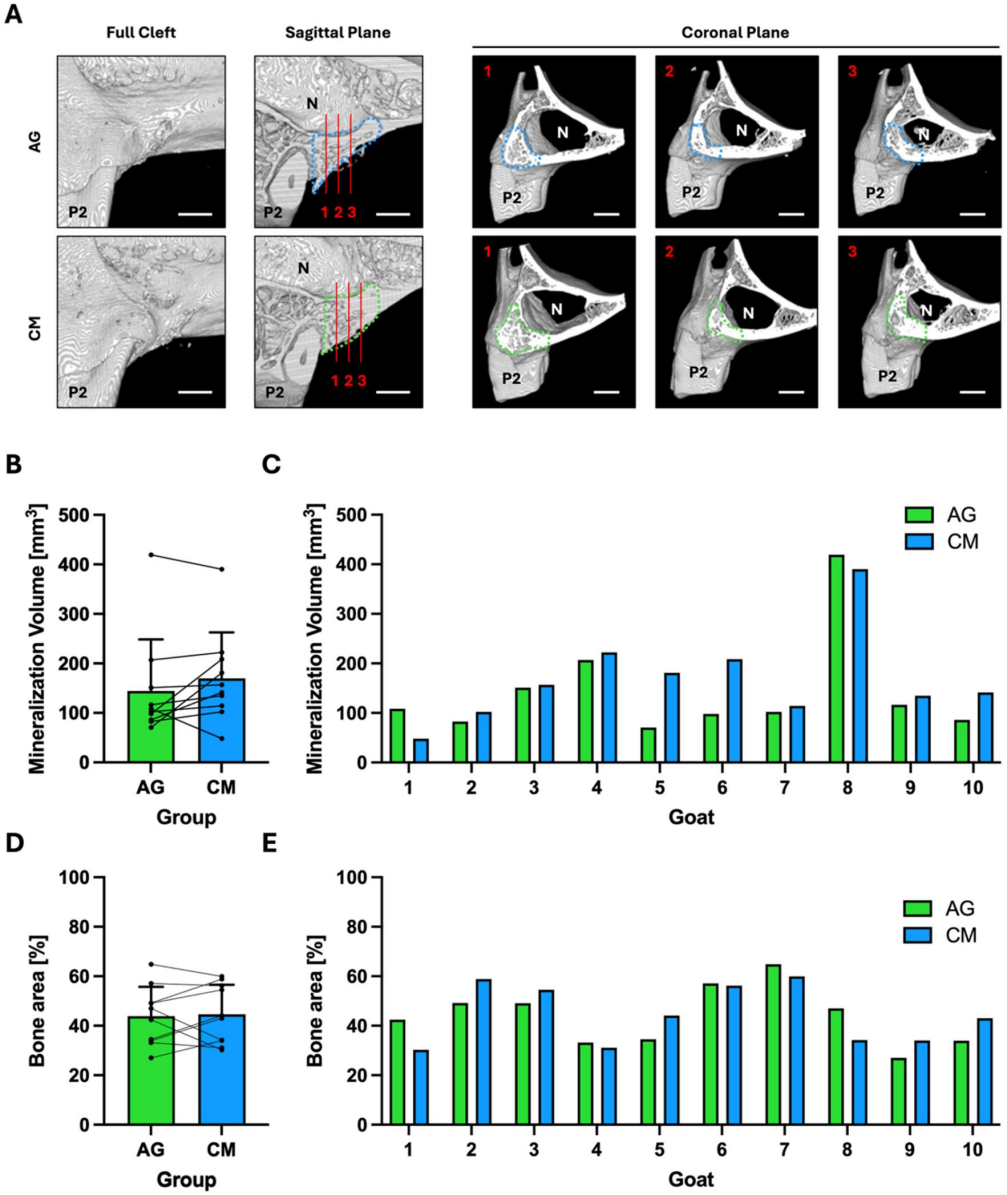
Mineralization and bone formation in the alveolar cleft defect. (**A**) Representative 3D microCT reconstructions from goat 7, selected as an example in which autograft (AG)- and soft callus mimetic (CM)-treated defects showed comparable outcomes, consistent with the overall absence of systematic treatment-related differences after 3 months *in vivo*. Reconstructions are shown as full defect, sagittal and coronal views. The defect region is outlined by dashed lines; solid lines mark planes of sectioning. P2 = second premolar; N = nasal cavity. Scale bar: 5 mm. (**B**) Quantification of mineralized tissue volume (microCT) within the defect region (individual animals shown as paired data points; mean ± SD; *N* = 10). (**C**) Mineralized tissue volume presented separately for each goat. (**D**) Quantification of bone tissue area within the defect region on histological sections, expressed as a percentage of the total defect area (individual animals shown as paired data points; mean ± SD; *N* = 10). Only mineralized bone was included; marrow spaces were excluded. (**E**) Bone tissue area displayed separately for each goat.

MicroCT analysis confirmed mineralized tissue formation in all defects across both treatment groups 3 months post-implantation. Except for the CM-treated site in goat 1 (Supplementary Figure S4), complete bony bridging was observed in all animals and both groups (Figure 2A; Supplementary Figure S5). Quantitative analysis showed no significant difference (paired one-sided t-test) in mineralized volume between AG- and CM-treated sites. Mean mineralized volume was 144.2 ± 104.4 mm^3^ in AG-treated sites and 170.0 ± 92.9 mm^3^ in CM-treated sites (Figure 2B). Individual responses varied among animals, with some showing greater regeneration in CM- and others in AG-treated sites (Figure 2C). Variability was more pronounced between animals than between treatments within the same animal. This likely reflected differences in initial defect size, anatomy and cleft morphology. Because of the bilateral split-mouth design, both defects within each animal were assumed to have comparable size and shape. A two-way ANOVA confirmed that the principal source of variance was attributable to inter-animal differences (90.8% of total variance), whereas treatment had no significant effect (1.9%; Supplementary Figure S6). These findings indicate that outcome variability arose predominantly from animal-specific factors rather than from the treatment itself.

#### Bone architecture and histology

Histomorphometric analysis confirmed consistent bone formation across all animals and treatment groups, in agreement with microCT findings. Bone volume fraction (bone area/total defect area) showed no significant difference (one-sided paired t-test) between AG- and CM-treated sites (43.85 ± 11.87% vs. 44.63 ± 11.94%; Figure 2D), indicating no treatment-related differences in the extent of bone fill. Responses varied among individual animals, with some showing higher BV/TV in CM- and others in AG-treated sites (Figure 2E). Variability was more pronounced between animals than between treatments within the same animal, likely reflecting differences in initial defect size, anatomy and cleft morphology. A two-way ANOVA confirmed that the principal source of variance was attributable to inter-animal differences (86.5% of total variance), whereas treatment had no significant effect (0.1%; Supplementary Figure S6). These findings indicate that outcome variability arose predominantly from animal-specific factors rather than from the treatment itself.

**Figure 3:**
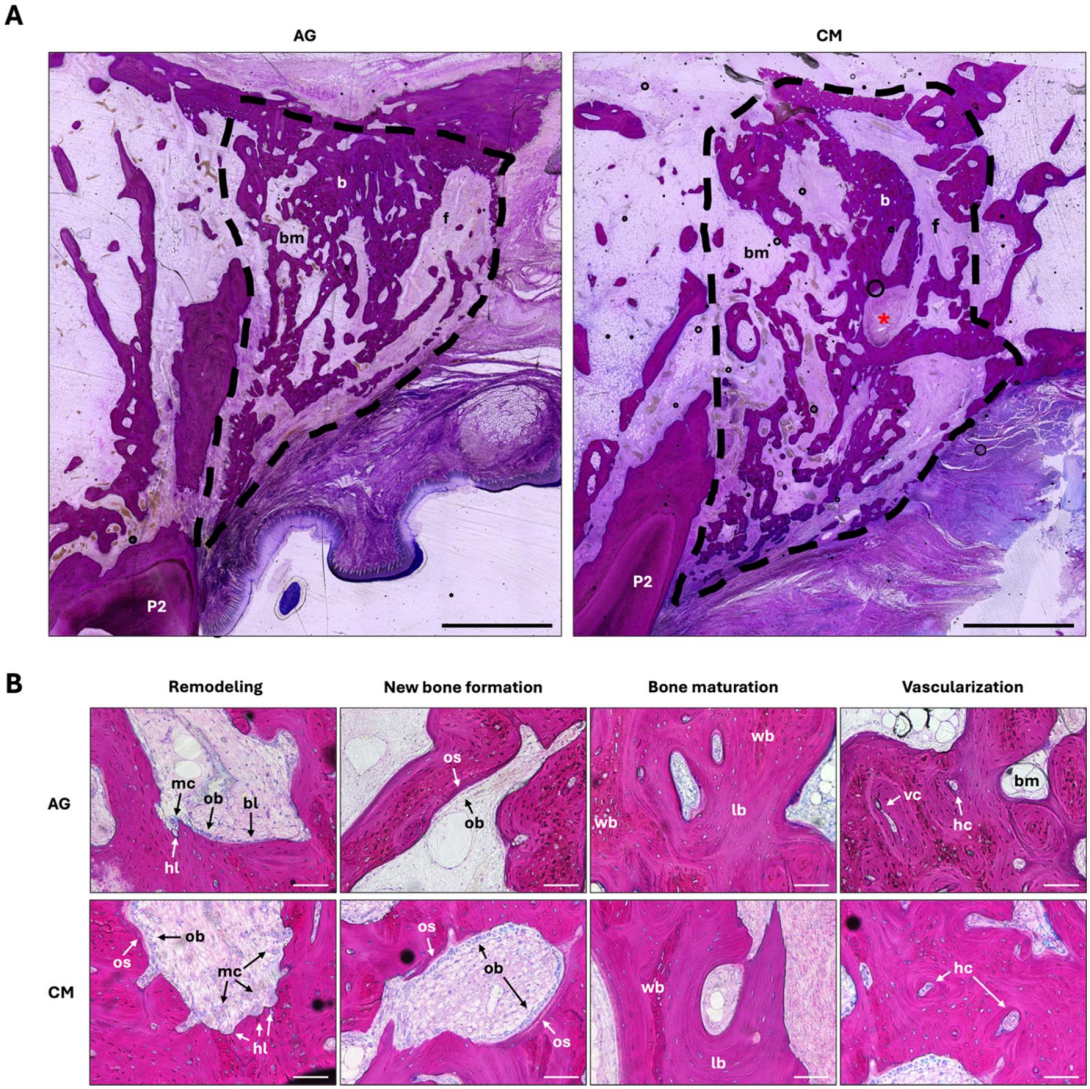
Histological evaluation of bone regeneration in alveolar cleft, comparing autograft and CM-treated defects after 3 months in goats. (**A**) Representative methylene blue and basic fuchsin-stained sections (goat 7) of autograft (AG) and soft callus mimetic (CM) groups after 3 months *in vivo*, showing bone regeneration across the full defect. Bone = bright pink; fibrous tissue = light pink; soft tissue = blue/purple. P2 = second premolar; f = fibrous tissue; b = bone; bm = bone marrow. * = putative remnants of CMs. Scale bars: 5 mm. (**B**) Higher magnification views from the central trabecular region of the regenerated defect in AG- and CM-treated sites of goat 7, illustrating key features of bone regeneration. mc = multinucleated cells; hl = howship’s lacunae; ob = osteoblasts; os = osteoid; bl = bone lining cells; wb = woven bone; lb = lamellar bone; vc = volkmann’s canal; hc = haversian canal; bm = bone marrow. Scale bars: 100 µm.

Although bone fill was evident throughout the defects, a continuous cortical bone layer had not yet formed in all animals. In representative sections, a thicker and more compact bone layer was observed along the superior defect margin, suggestive of early cortical bone formation, whereas the central region predominantly consisted of trabecular bone with immature woven or plexiform morphology (Figure 3A). MicroCT scans corroborated the presence of a thin cortical layer, although it remained less developed than the native cortical bone above the second premolar (Supplementary Figure S7). The degree of cortical thickening and the extent of trabecular bone fill varied among animals, with no systematic differences between AG- and CM-treated groups. These differences appeared to be animal-specific (Supplementary Figure S8).

Between trabeculae, both bone marrow and fibrous tissue were present (Figure 3A). Their distribution varied between animals but remained consistent between contralateral sites within individual goats (Supplementary Figure S8). In CM-treated sites, occasional ring-shaped fibrous regions suggested possible CM remnants (Figure 3A), whereas in AG-treated sites, areas of lamellar bone with abnormal cellular morphology were morphologically consistent with non-remodeled autograft remnants. This interpretation was further supported by fluorochrome labeling. Calcein green labeling was absent in these regions but present in adjacent newly formed bone at integration zones (Supplementary Figure S9).

Evidence of active bone remodeling was observed in both treatment groups. Multinucleated cells located within resorption pits (Howship’s lacunae) indicated osteoclastic activity, whereas osteoblasts with cuboidal morphology were aligned along osteoid seams (Figure 3B). Bone-lining cells were also present on mineralized bone surfaces. Most regenerated bone exhibited an immature woven architecture with high osteocyte density. However, regions with early lamellar organization were also evident, suggesting progressive tissue maturation. Structures resembling Haversian channels and Volkmann-like canals were observed throughout the regenerated regions, together with adjacent marrow spaces (Figure 3B). These features suggest tissue organization typically associated with vascularized bone.

#### Bone formation dynamics

Sequential fluorochrome labeling confirmed ongoing bone formation and remodeling in all animals across both treatment groups. Both calcein green (CG; 1 month) and oxytetracycline (OTC; 2 months) signals were detected in all goats and in all defects.

**Figure 4:**
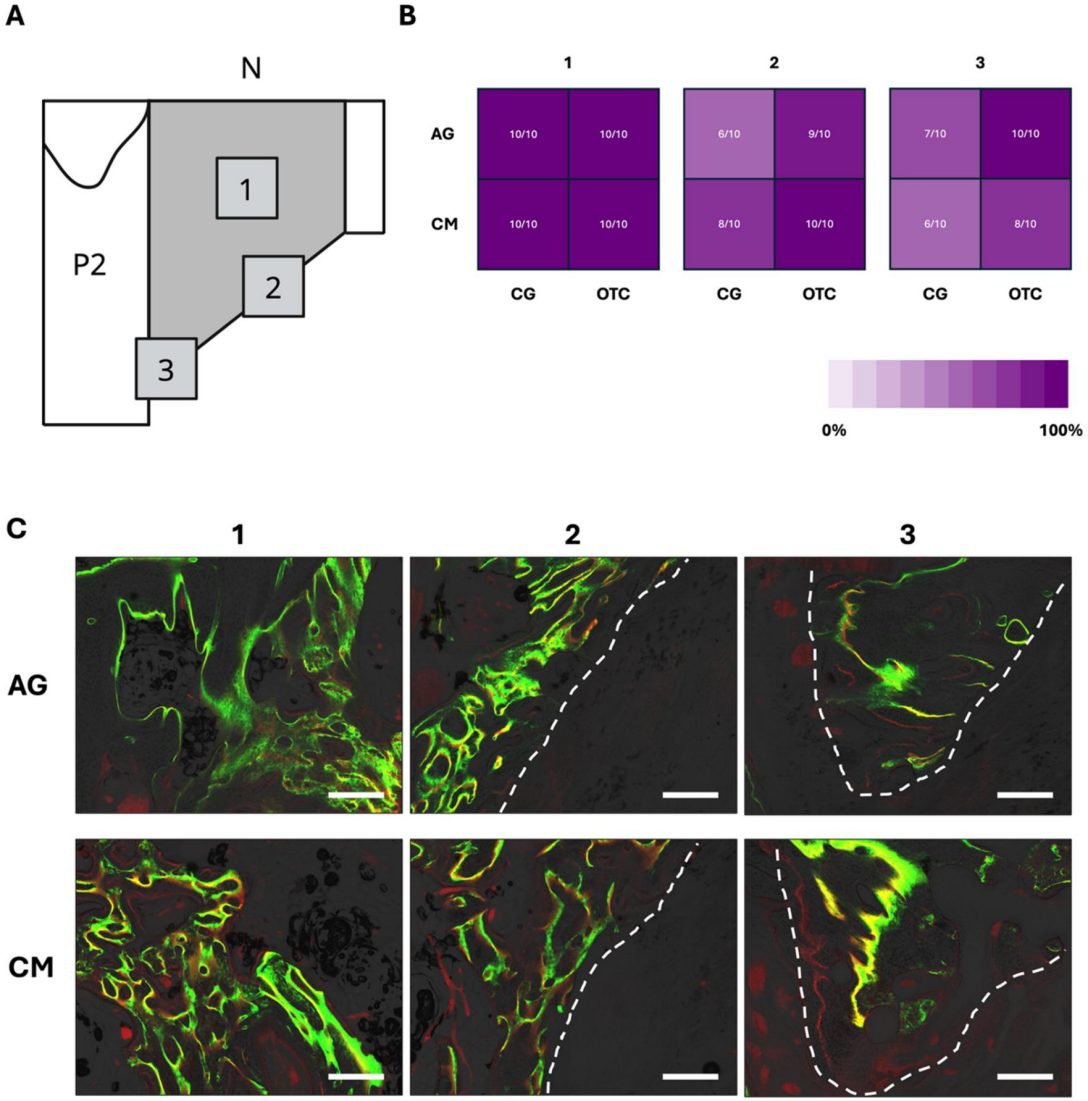
Bone formation dynamics assessed by sequential fluorochrome labeling. (**A**) Schematic illustration of the three standardized regions analyzed within each defect: center (1), side edge (2), and bottom edge (3) relative to the second premolar (P2) and the nasal cavity (N). (**B**) Heat maps summarizing the proportion of goats meeting the ‘fully labeled bone’ criterion for calcein green (CG; 1 month) and oxytetracycline (OTC; 2 months) labeling across regions and treatment groups. Darker shades indicate a higher frequency of ‘fully labeled bone’ outcomes. (**C**) Representative images (goat 6) illustrating fluorochrome incorporation patterns in areas 1–3. Dashed white lines delineate defect boundaries. Scale bars: 100 µm.

For each defect, three standardized regions (center (1), side edge (2), and bottom edge (3)) were analyzed (Figure 4A). As described in the ‘Methods’ section, CG and OTC labeling were scored separately in each region based on whether the fluorochrome signal was distributed throughout the bone area. Heat maps summarizing these outcomes showed that all goats in both treatment groups met the ‘fully labeled bone’ criterion for both fluorochromes in the central region, consistent with early initiation and sustained remodeling activity in this zone. At the side and bottom edges, fully labeled bone was less frequent, particularly for OTC. This indicates that parts of the bone in these regions had formed after the second labeling time point or were still undergoing active remodeling. The distribution of labeling patterns did not differ between AG- and CM-treated sites (Figure 4B).

Representative images from one goat illustrate these patterns, with CG and OTC labeling distributed throughout the bone compartment in regions 1, 2 and 3 on both sides, except for region 3 in the CM‑treated site, where only CG fully labeled the bone area (Figure 4C). This local absence of OTC is consistent with continued bone formation beyond the second labeling time point and was also observed in AG‑treated regions in other animals, in line with the overall analysis showing no systematic differences in labeling patterns between treatments.

### Long-term cryostorage of soft callus mimetics

**Figure 5:**
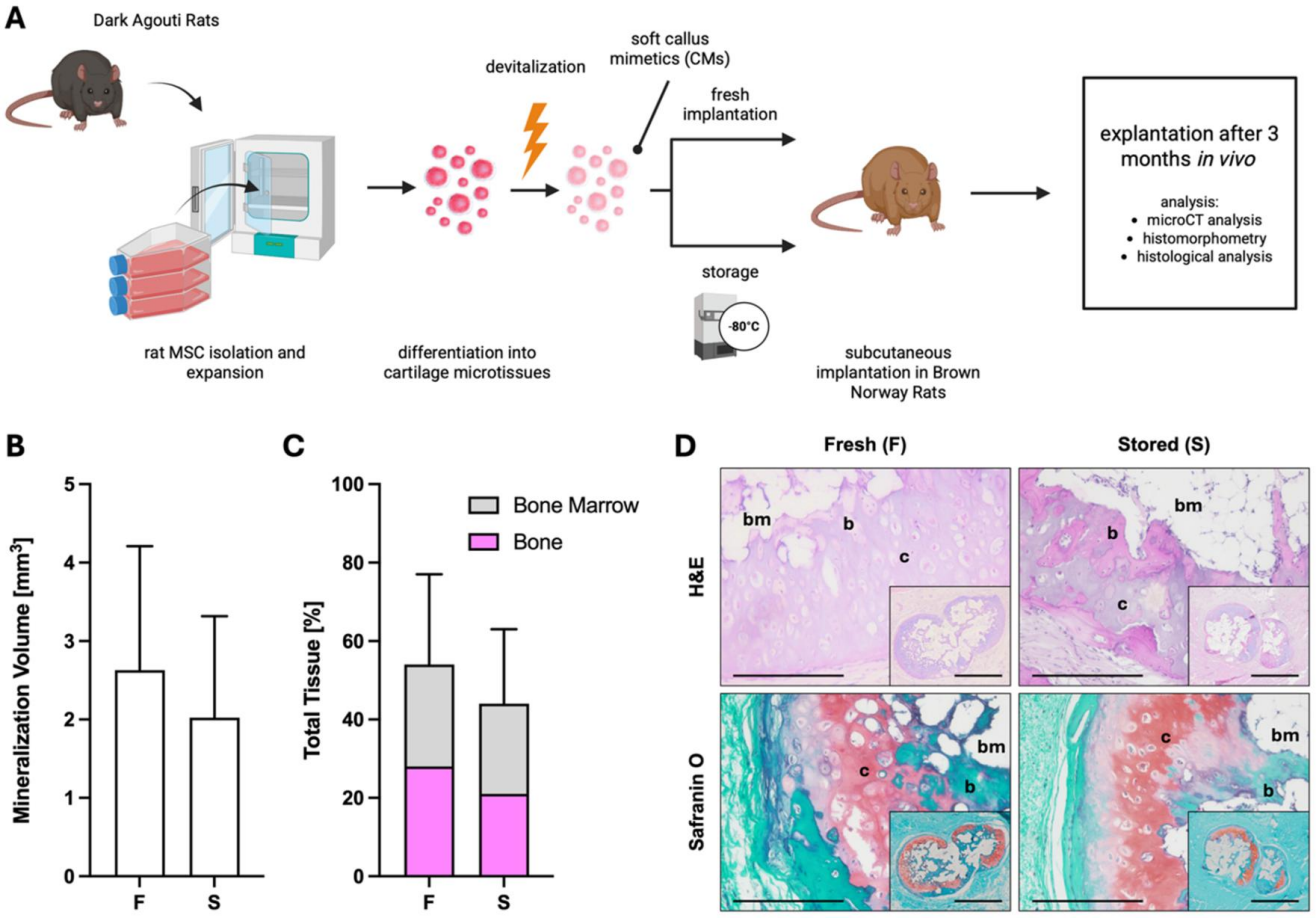
Osteoinductive potential of stored (S) soft callus mimetics (CMs) compared with previously reported fresh (F) CMs. (**A**) Schematic overview of the rat subcutaneous implantation study, in which allogeneic rMSC‑derived CMs, either previously implanted fresh (F) [20] or following prolonged dry storage at −80°C for 9 months (S), were implanted subcutaneously. They were analyzed 12 weeks after implantation. (**B**) MicroCT quantification of mineralized tissue volume in explants after subcutaneous implantation in allogeneic rats. Data are presented as mean ± SD. N(F) = 6. N(S) = 9. (**C**) Histomorphometric analysis of bone and bone marrow in explants. Data are presented as mean ± SD of combined total tissue. N(F) = 6. N(S) = 9. (**D**) Representative H&E and safranin O/fast green–stained sections showing new bone (b), bone marrow (bm), and cartilage (c). Insets depict entire explant cross-sections. Scale bars: 100 μm (main) and 1 mm (insets). No significant differences were found in B and C.

In a subcutaneous implantation model in rats, the osteoinductive capacity of CMs stored dry for 9 months at −80°C was compared with previously reported fresh CM data obtained in the same model [20]. All CMs were generated in the same production run. In the previous study, part of the freshly prepared CMs was implanted directly, whereas the remaining CMs were stored and implanted in the present study after 9 months. The stored CM group was implanted under matched conditions with respect to collagen carrier, implantation site, recipient rat strain, implantation duration, and analytical endpoints; however, fresh and stored CMs were not implanted concurrently. Bone formation at 12 weeks was quantified by microCT, histology and histomorphometry. An overview of the experimental workflow is depicted in Figure 5A.

MicroCT analysis showed that mineralized tissue volume in stored CMs (2.02 ± 1.29 mm³) was comparable to that previously reported for freshly implanted CMs (2.63 ± 1.58 mm^3^) (Figure 5B). Histomorphometric analysis likewise revealed similar total bone fractions in stored and previously reported fresh CMs (44.89 ± 19.34% vs. 54.34 ± 23.12%; Figure 5C). Histological evaluation with H&E and Safranin O/Fast Green confirmed analogous tissue architecture, including new bone, bone marrow, and residual cartilage in both stored CMs (this study) and fresh CMs from the prior work (Figure 5D). In both groups, newly formed bone was observed in close association with residual cartilaginous matrix. Marrow-like tissue was present within intertrabecular regions. Together, these findings are consistent with partial remodeling of the CM template into bone- and marrow-containing tissue. Residual Safranin O-positive cartilage suggested that CM remodeling was ongoing at the 12-week endpoint.

## Discussion

This study demonstrates that CMs can support bone regeneration comparable to autologous bone grafts in a large animal maxillofacial model. Rather than acting as a passive filler, the CM implants appeared to support bone formation through a cartilage-template-driven process. This suggests that endochondral cues retained within the devitalized matrix can remain instructive in a maxillofacial environment. Importantly, mineralized tissue in CM-treated defects was newly generated after implantation, whereas the autograft group contained both newly formed bone and residual non-remodeled grafted bone. This distinction is relevant when interpreting comparable bone volumes between groups, as the CM group did not contain implanted mineralized tissue at baseline. Together, these findings support the feasibility of an endochondral approach for maxillofacial bone repair in large mammals.

To our knowledge, this is the first large animal maxillofacial study demonstrating bone regeneration from a pre-formed cartilage-based template. This finding extends the anatomical applicability of endochondral bone regeneration. It shows that endochondral matrix-derived cues can remain instructive even in maxillofacial bone, which normally forms and heals predominantly through intramembranous processes. This is relevant because maxillofacial bones differ from long bones in developmental origin, resident progenitor populations and healing context [11].

While both vital and engineered cartilage tissues can, in principle, initiate this cascade through the release of hypertrophic and angiogenic factors [24–26], the current findings highlight that such endochondral signaling can be preserved even after devitalization [5, 6]. Mechanistically, this regenerative behavior likely arises from matrix-retained molecular cues within the CMs (including VEGF, COL10A1, MMP13 and ALP) that collectively orchestrate vascular invasion, resorption, and mineral deposition [27–31]. These molecules are well-established mediators of hypertrophic cartilage remodeling and bone coupling during endochondral ossification [2, 32, 33]. Their persistence within the devitalized extracellular matrix may recreate a transient endochondral microenvironment. This microenvironment may coordinate scaffold degradation with bone deposition, even in tissues that do not typically form cartilage intermediates. This ability distinguishes CMs from most synthetic ceramics or polymer scaffolds, which rely primarily on osteoconduction and lack coupled remodeling and vascular integration [34].

The bilateral goat alveolar cleft model provided a stringent and anatomically relevant platform to test this hypothesis. Although originally developed for alveolar cleft reconstruction [24, 35, 36], the model was adopted here to serve as a representative maxillofacial defect for evaluating EBR within an intramembranous environment. Each animal received both treatments. A CM was implanted in one cleft, and an autologous bone graft was placed in the contralateral cleft. This split-mouth design enabled direct within-animal comparison. This approach avoids comparisons with inter-animal variability arising from systemic, metabolic, or anatomical/geometric factors, thereby increasing statistical power and reducing the number of animals required [22, 37, 28]. Goats offer a suitable defect size, bone turnover rates, and maxillofacial anatomy to approximate human conditions [37, 39], making them an appropriate large animal platform for translational assessment of bone regeneration strategies. Thus, the model served as a clinically relevant testing platform for CM performance rather than as the primary focus of the study.

Several considerations arise when envisioning the potential use of CMs for clinical cleft repair. First, the defects created in this study were acute rather than chronic, representing a simplified biological environment compared with long-standing alveolar clefts that often include scarred soft tissues and altered vascularization [40–42]. Second, the defect type is not of critical size, which was appropriate for this study setup where the aim was to demonstrate feasibility in large animals and compare to gold standard clinical treatment. Third, the model did not include oronasal fistula closure or combined soft-tissue reconstruction, both of which are critical components of surgical cleft repair that could influence graft integration [40, 41, 43]. Fourth, the three-month follow-up captured the main phase of early bone formation and remodeling but did not encompass long-term maturation, load adaptation or dental eruption dynamics [42]. These parameters will be important for evaluating long-term functionality in an authentic cleft setting. Finally, only female goats were included. This placed both CMs and autografts in a hormonally dynamic context, where sex hormones may influence bone turnover and healing kinetics [44, 45]. Demonstrating comparable performance of CMs and autografts under these conditions provides a conservative test of efficacy, although future studies including both sexes will be needed to confirm that regenerative outcomes are consistent across biological variability.

Additional study-specific limitations should be considered. The goat CMs were generated from two allogeneic donor cell populations. Donor-related differences in matrix distribution, construct size, and GAG content were observed before implantation, highlighting the importance of characterizing CM batches prior to *in vivo* use. Because the number of available cells and resulting constructs was limited, CM1 and CM2 were combined at a 1:1 ratio to generate a single pooled allogeneic implant formulation, and separate CM1-only and CM2-only groups were not included. Therefore, the present study was not designed to determine how donor variability or donor pooling affects *in vivo* performance. Future studies will be needed to establish donor-release criteria and assess batch-to-batch consistency. In addition, a defect-only negative control was not included. Although the defect used in this study may not represent a strict critical-size defect, complete spontaneous closure within the 3-month follow-up period would not be expected. Nevertheless, the absence of a defect-only control prevents direct quantification of spontaneous healing in this specific experimental setting. Finally, no mechanical testing, functional loading assessment, dental eruption analysis, or long-term follow-up was performed. Therefore, the present data support biological bone regeneration and tissue integration after 3 months, but do not yet establish functional equivalence to autograft for clinical maxillofacial reconstruction.

While the present work establishes proof-of-concept for CMs in a demanding large animal maxillofacial defect, routine alveolar cleft repair may not represent the primary clinical niche for this technology, as autografts can often be replaced by simpler and less costly substitutes in this indication. Instead, CMs may be most valuable for complex maxillofacial or orthopedic defects with large volumes, compromised biology, or limited conventional graft options, rather than for routine cleft repair [34].

From a translational perspective, CMs unite biological potency with practical manufacturability. Constructs cryostored for up to nine months at −80°C retained substantial *in vivo* bone-forming potential when compared with previously reported freshly prepared lots. The fresh CM data were obtained from a previous experiment rather than from a concurrent control group. Therefore, these findings should be interpreted as evidence that prolonged frozen storage did not abolish osteoinductive capacity. They should not be interpreted as definitive proof of equivalence between fresh and stored constructs. This frozen stability supports scalable, centralized manufacturing with controlled quality assurance and lot release. The devitalized nature of the implants further simplifies regulatory pathways, as devitalized CMs may be regulated differently from living-cell therapies, although formal classification will depend on jurisdiction and manufacturing specifics [46–50]. Their favorable handling characteristics, being malleable yet cohesive and able to conform to defect geometry, further enhance surgical usability.

Compared with current clinical standards such as β-TCP or demineralized bone matrix (DBM), which provide reliable osteoconduction but limited capacity to recapitulate developmental remodeling, CMs offer an intrinsically pro-regenerative matrix that activates endochondral healing pathways [34, 51]. In contrast to recombinant growth-factor-based or living-cell-based therapies, CMs deliver osteoinductive potential in a devitalized format. This decouples their biological activity from the regulatory and safety challenges associated with administering viable cells or high-dose growth factors [52–54]. However, before CMs can be positioned as an alternative to autograft, future studies should establish donor-release criteria, storage-release potency assays, mechanical competence of the regenerated tissue, longer-term remodeling, and performance in clinically more challenging defects. Together, these attributes position CMs as a promising storable biomaterial platform for further development across both maxillofacial and orthopedic indications.

## Conclusion

This study shows that devitalized, endochondral-inspired callus mimetics can support bone regeneration in a large animal maxillofacial defect model. In the goat study, CM-treated defects showed no evidence of inferior early structural outcomes relative to autologous bone grafts. This was observed across imaging, histomorphometry, histology, and fluorochrome-based remodeling assessment. These findings support the feasibility of this approach in an intramembranous environment. In the rat study, CMs retained osteoinductive capacity after 9 months of cryostorage in comparison with previously reported fresh controls implanted under matched but non-concurrent conditions. Together, these data support further development of CMs as a cryostorable biomaterial platform for bone regeneration, while future studies should address donor variability, long-term remodeling, and mechanical and functional performance in more clinically challenging defects.

## Supplementary Material

rbag125_Supplementary_Data
