# Supplementary material for: Cryostorable callus mimetics support endochondral bone regeneration in a large-animal maxillofacial defect
Source: Regen Biomater. 2026 Jun 10;13:rbag125. doi: 10.1093/rb/rbag125 (PMC13332429; doi:10.1093/rb/rbag125)
Supplement: rbag125_Supplementary_Data [file rbag125_supplementary_data.docx]

**Supplementary data**

**
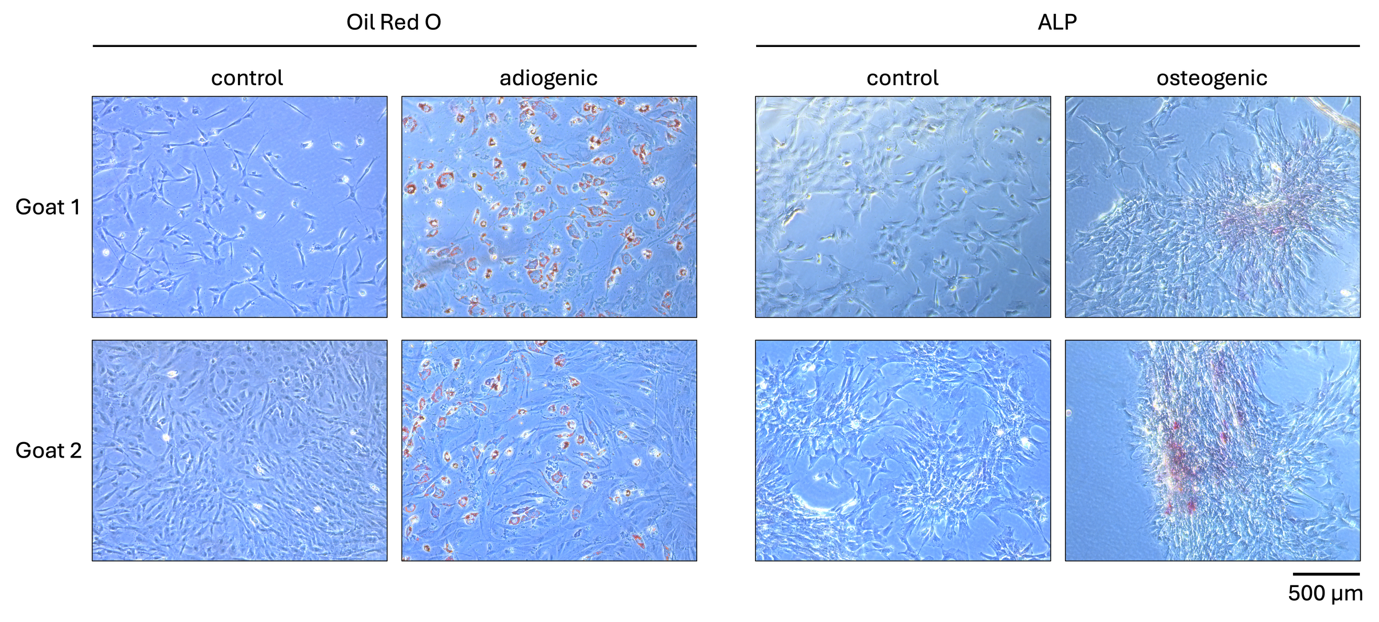
Supplementary Figure 1: Adipogenic and osteogenic differentiation potential of goat MSC donor populations.** Representative images showing adipogenic (Oil Red O staining) and osteogenic (ALP staining) differentiation of the two goat MSC donor-derived cell populations used to generate CM1 (goat 1) and CM2 (goat 2). Corresponding controls (cultured in expansion medium) were also stained.


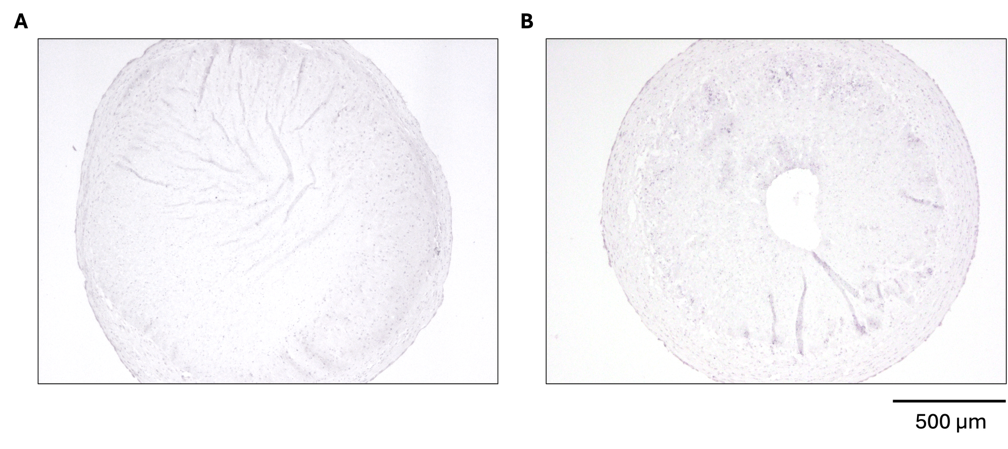


**Supplementary Figure 2: Isotype controls for collagen II and VEGF immunostaining in goat soft callus mimetics.** Representative sections of goat soft callus mimetics stained with matched mouse isotype controls corresponding to the immunostaining conditions used for (A) collagen II and (B) VEGF. No specific staining was observed, confirming the specificity of the collagen II and VEGF immunostaining shown in Figure 1B.

**
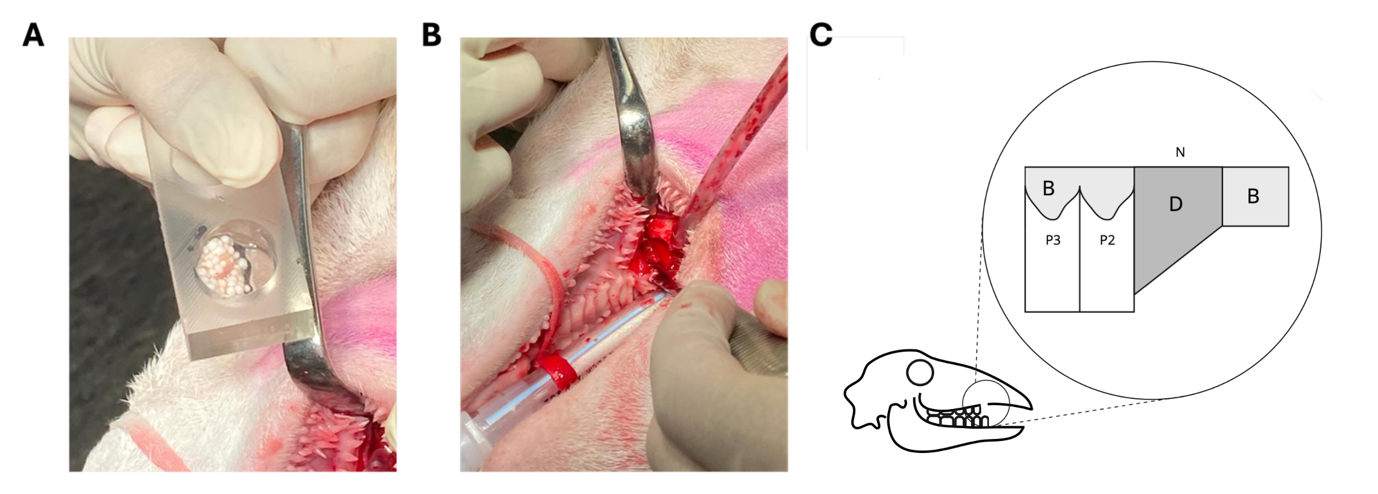
**

**Supplementary Figure 3: Soft callus mimetic (CM) implantation and defect overview.** (A) Soft callus mimetics (CMs) embedded within the fibrin carrier prior to implantation (goat 2). (B) Intraoperative image showing the created defect site. (C) Schematic representation of the defect location and geometry. D = defect site. N = nasal cavity. P2 = second premolar. P3 = third premolar. B = bone.


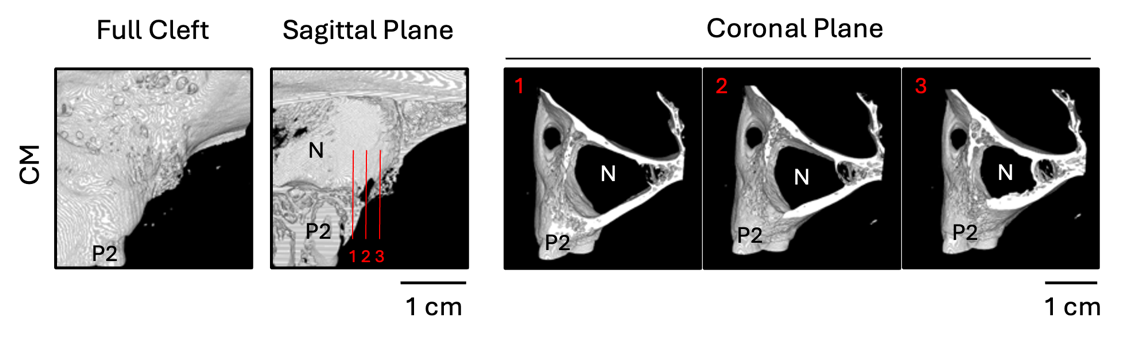


**Supplementary Figure 4: Unbridged CM-treated defect site in goat 1.** 3D microCT reconstructions of the soft callus mimetic (CM) group after 3 months *in vivo*, shown as full defect, sagittal, and coronal views. Red lines mark planes of sectioning. P2 = second premolar; N = nasal cavity.


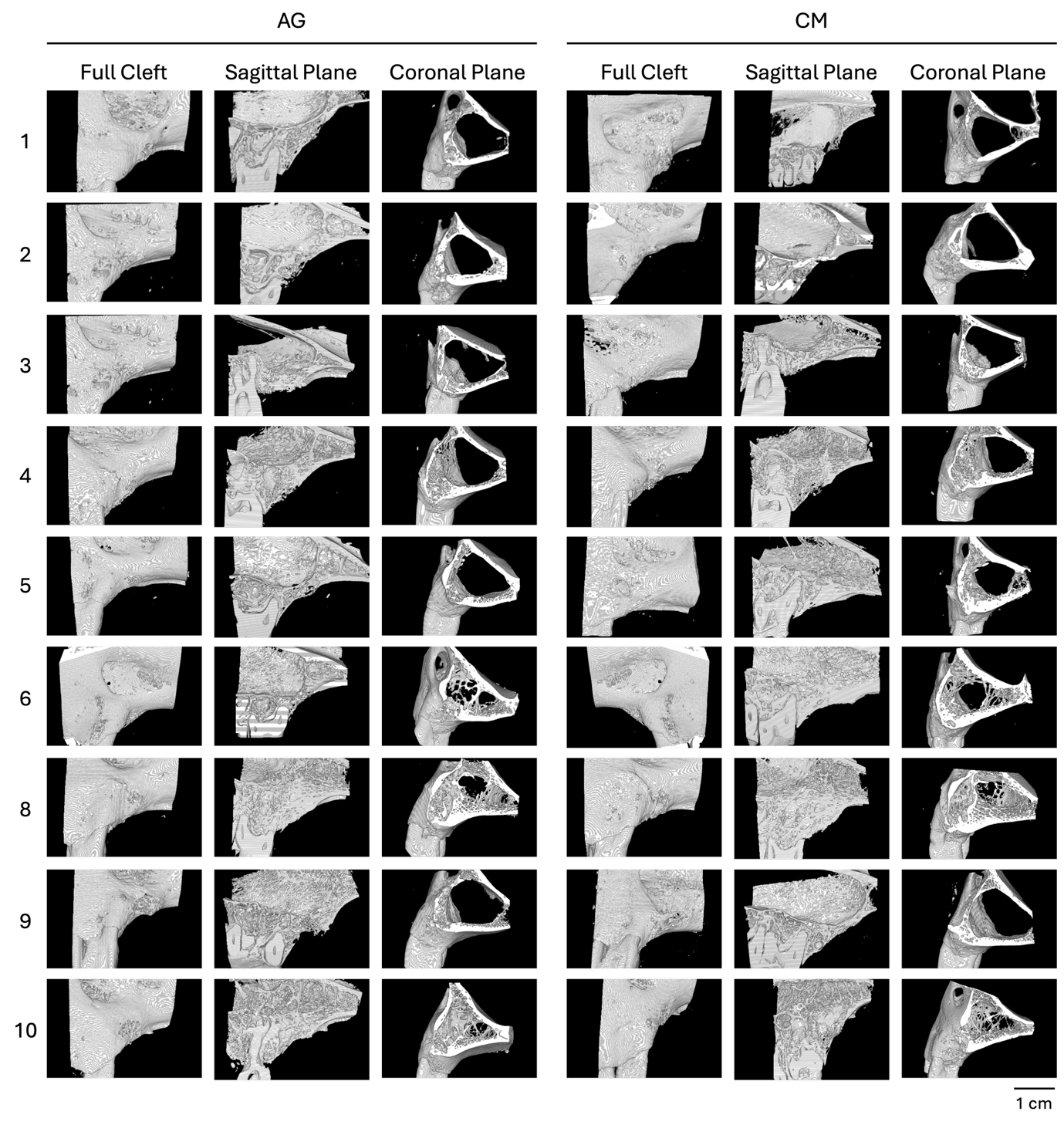


**Supplementary Figure 5: Mineralization in all goat cleft defects.** 3D microCT reconstructions of autograft (AG) and callus mimetic (CM) groups after 3 months *in vivo*, shown as full defect, sagittal, and coronal views. Goat 7 is not shown, as it is shown in the main text.

**
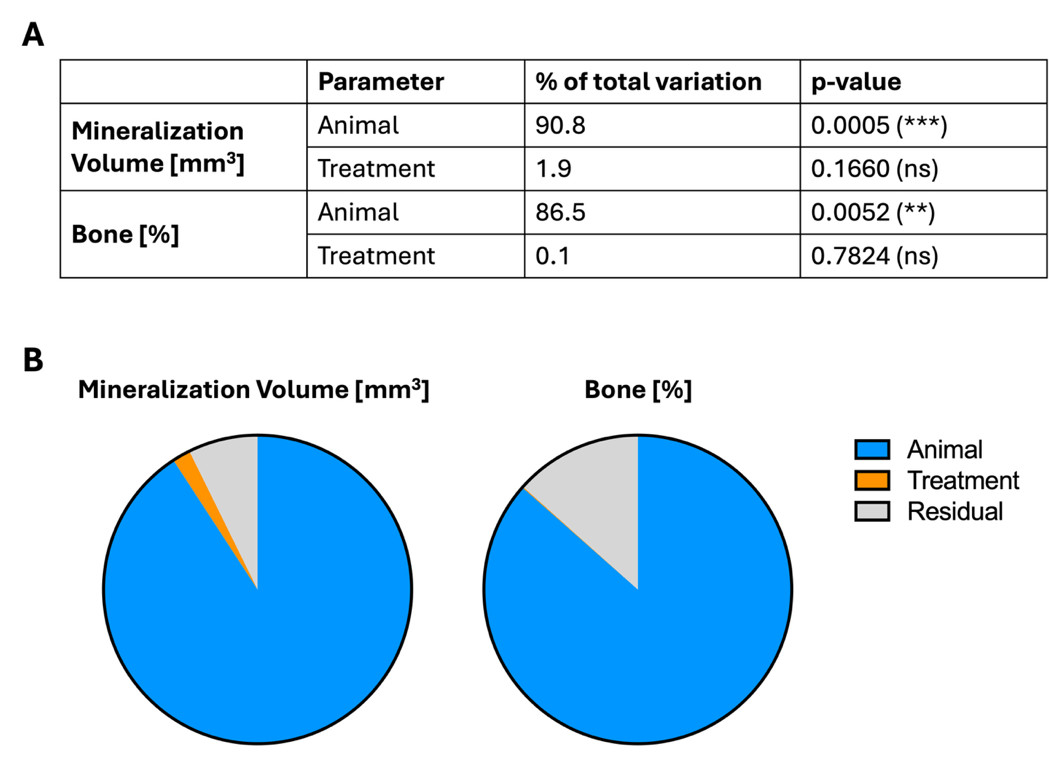
**

**Supplementary Figure 6: Contribution of animal and treatment effects to variability in mineralized tissue and bone outcomes.** (A) Table summarizing the percentage of total variance in mineralized volume and bone percentage attributable to animal identity and treatment, together with corresponding p-values from two-way ANOVA. (B) Pie charts illustrating the relative contributions of animal, treatment, and residual variance components for mineralized volume (left) and bone percentage (right).

**
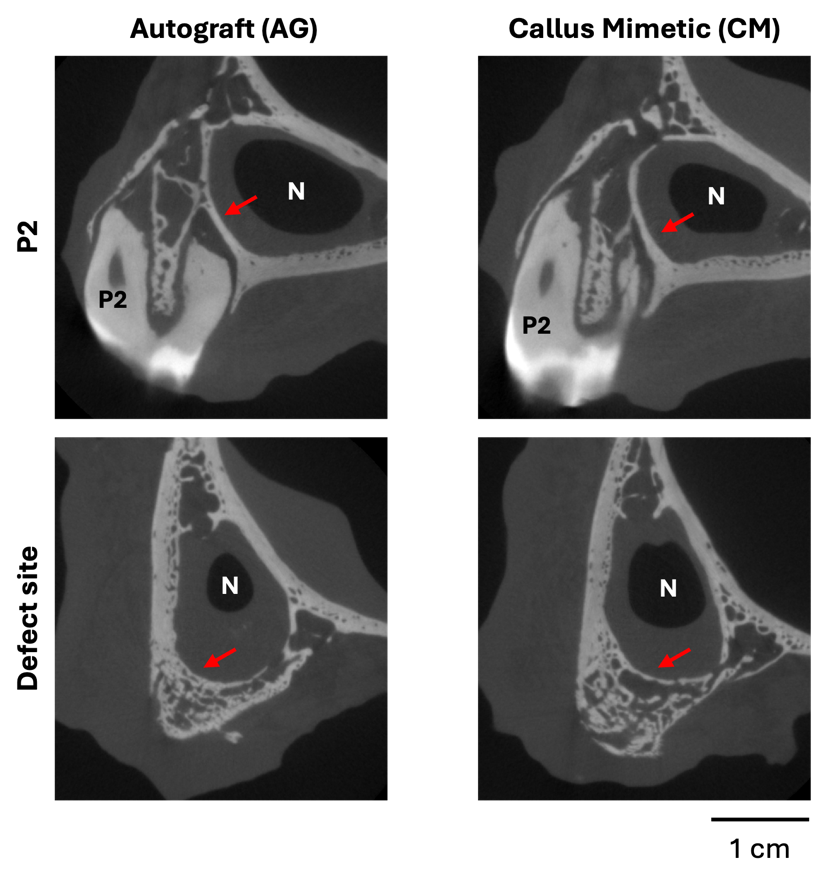
**

**Supplementary Figure 7: 2D microCT scans.** Autograft (AG) and callus mimetic (CM) groups after 3 months *in vivo* (goat 7) are shown. Coronal views at the center of the second premolar (P2) and at the center of the defect site are shown. Red arrows point at cortical bone. N = nasal cavity.


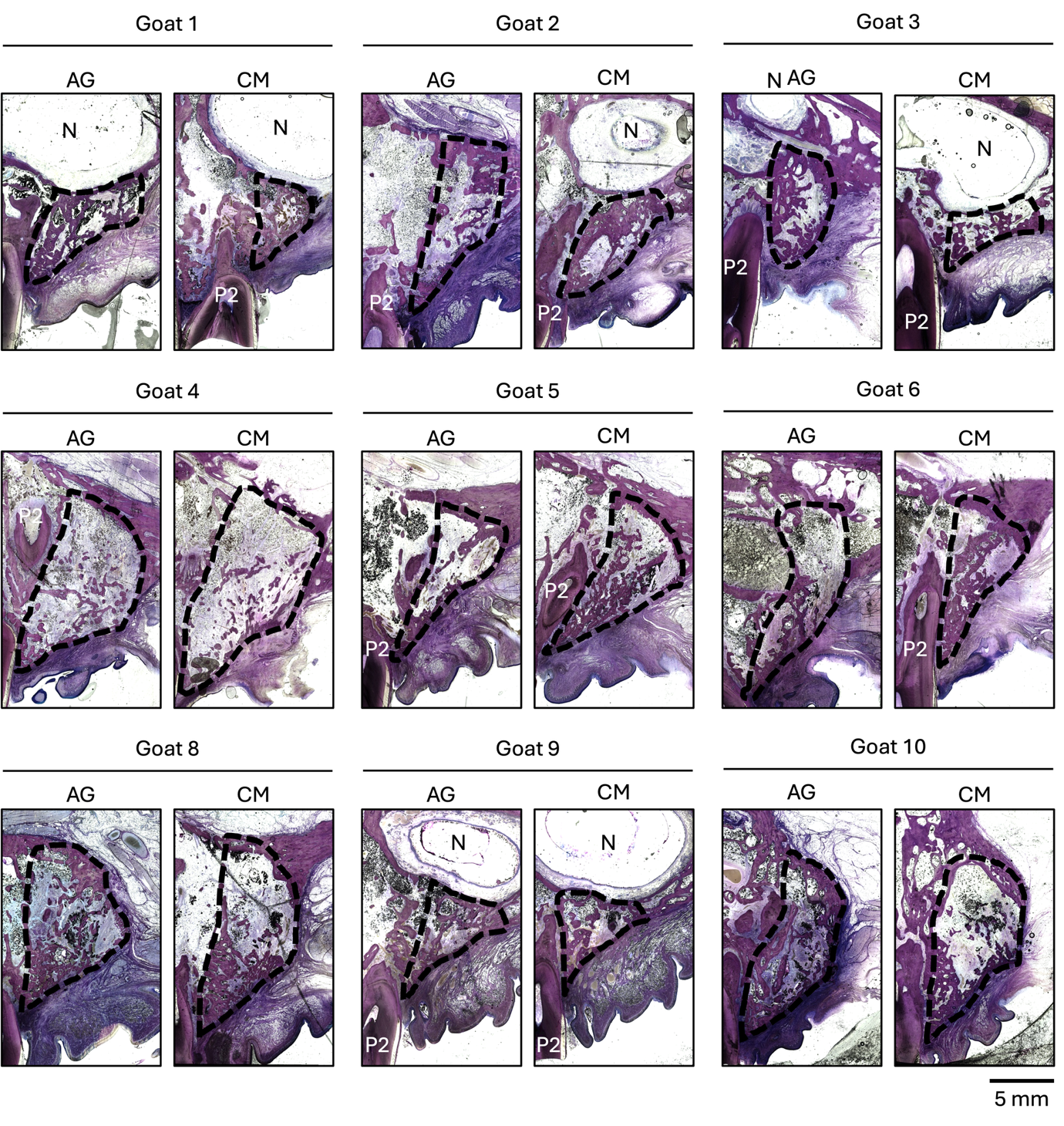
**Supplementary Figure 8: Bone formation in cleft defects.** Methylene blue/basic fuchsin staining of autograft (AG) and callus mimetic (CM) groups after 3 months *in vivo* (central sagittal plane). Dotted black line indicates border of defect area. Bone = bright pink; fibrous tissue = light pink; soft tissue = blue/purple; bone marrow/marrow spaces = white. Dark material occasionally present within marrow or defect spaces may represent blood, tissue remnants, or processing-related artifact. P2 = second premolar; N = nasal cavity. Goat 7 is not shown, as it is shown in the main text.


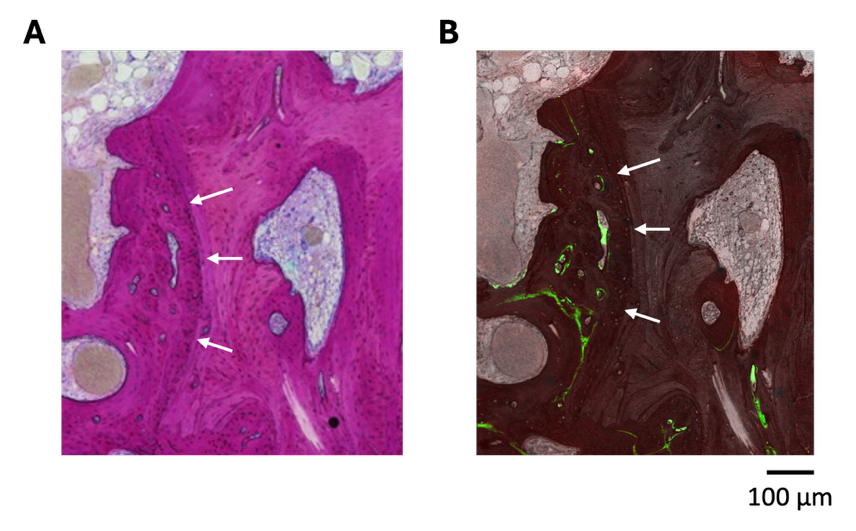


**Supplementary Figure 9: Presence of non-remodeled grafted bone in the autograft group.** (A) Methylene blue/basic fuchsin-stained MMA section of an autograft-treated defect (goat 6) showing the interface between newly formed woven bone and a region morphologically consistent with non-remodeled grafted bone. (B) Fluorochrome imaging of the same section enabled direct alignment with the histological image. Calcein green labeling was detectable in newly mineralizing bone but absent from the adjacent grafted bone region. Oxytetracycline labeling was not sufficiently intense to be detected after staining. Together with the distinct morphology of this region, the absence of calcein green signal supports its interpretation as pre-existing, non-remodeled grafted bone rather than newly formed bone. White arrows indicate the interface between newly formed bone and putative non-remodeled grafted bone.
